# Error in Description of Limitations

**DOI:** 10.1001/jamanetworkopen.2021.23159

**Published:** 2021-07-23

In the Invited Commentary titled “Surfactant Treatment—a National, Population-Based Study of Adherence to Best Practice, Off-Label Use, and Associations With Outcomes,”^1^ published May 5, 2021, it was incorrectly stated that the study by Challis et al^2^ did not report data on oxygen requirement at 36 weeks postmenstrual age. This article has been corrected.^1^

## References

[1] Ramanathan R. Surfactant treatment—a national, population-based study of adherence to best practice, off-label use, and associations with outcomes. JAMA Netw Open. 2021;4(5):e217848. doi:10.1001/jamanetworkopen.2021.7848 33950213

[2] Challis P, Nydert P, Håkansson S, Norman M. Association of adherence to surfactant best practice uses with clinical outcomes among neonates in Sweden. JAMA Netw Open. 2021;4(5):e217269. doi:10.1001/jamanetworkopen.2021.7269 33950208 PMC8100866

